# CRISPR/Cas9-mediated p53 and Pten dual mutation accelerates hepatocarcinogenesis in adult hepatitis B virus transgenic mice

**DOI:** 10.1038/s41598-017-03070-8

**Published:** 2017-06-05

**Authors:** Yongzhen Liu, Xuewei Qi, Zhenzhen Zeng, Lu Wang, Jie Wang, Ting Zhang, Qiang Xu, Congle Shen, Guangde Zhou, Shaomin Yang, Xiangmei Chen, Fengmin Lu

**Affiliations:** 1 Department of Microbiology, School of Basic Medical Sciences, Peking University Health Science Center, Beijing, 100191 P.R. China; 2 Department of Pathology and Hepatology, Beijing 302 Hospital, Beijing, 100039 P.R. China; 3 Department of Pathology, Peking University Health Science Center, Beijing, 100191 P.R. China

## Abstract

The p53 mutation and altered Pten expression are two most common genetic events in Hepatitis B virus (HBV) infection related hepatocellular carcinoma (HCC). To confirm the causative role of *p53* and *Pten* somatic mutation in HCC development, we established CRISPR/Cas9-mediated somatic gene disruption via hydrodynamic tail vein injection, allowing for *in vivo* targeting *p53* and *Pten* simultaneously in adult HBV transgenic mice. Here we demonstrated that the utility of this approach resulted in macroscopic liver tumors as early as 4 months’ post injection and most tumors harbored both *p53* and *Pten* loss-of-function alterations. Immunohistochemical (IHC) and histopathology analysis demonstrated that the tumors were positive for Glutamine synthetase (GS), a marker of HCC and accompanied with prominent lipid accumulation. The study here indicated that CRISPR/Cas9-mediated *p53* and *Pten* somatic mutation accelerated hepatocarcinogenesis in adult HBV transgenic mice. This method also provides a fast and convenient system for generating mouse model of HCC with HBV infection characteristics.

## Introduction

Hepatocellular carcinoma (HCC) is one of the most common liver cancer and becoming the second leading cause of cancer-related deaths in the world^1^. Persistent hepatitis B virus (HBV) infection is one of the major risk factors for HCC development, which accounts for more than 50% of HCC worldwide^2^. It has been suggested that the inflammation, liver damage, and regeneration induced by chronic HBV infection may foster the accumulation of genetic and epigenetic defects leading to cancer onset^3^. However, the mechanism of the hepatocarcinogenesis from the initial HBV infection remain to be elucidated.

There is now mounting experimental evidence indicating that the genomic features are significantly different between the HBV-related HCC and non-HBV-HCC. Compared with tumors associated with other risk factors including hepatitis C virus (HCV) infection, exposition to aflatoxin B1, alcohol consumption and metabolic diseases, HBV-related liver tumors have a higher rate (32–47%) of p53 inactive mutations^4–8^. Furthermore, p53 mutations were associated with shorter survival only in HBV-related HCC^9, 10^. These results suggested that genomic features were significantly different in the group of HBV-related HCC compared with non-HBV-HCC and p53 mutation may play an important role in the development of HBV-related HCC. In addition to p53 mutation, the inactivation of the phosphatase and tensin homolog (Pten) through genetic or post-translational modifications is found in about half of primary HCC patients^11, 12^. Liver-specific knockout of Pten in mice results in fatty liver disease and late-onset liver cancer^13, 14^. Therefore, loss of Pten function may also play a pivotal role in promoting carcinogenesis of HCC. Despite the fact that mutation/inactivation of p53 and Pten has been extensively studied and is implicated in HCC development, there is a lack of direct evidence supporting the role of p53 and Pten loss in initiating tumor formation in the liver during HBV infection.

CRISPR/Cas9 system, the newly developed genome editing tool consists of a single guide RNA (sgRNA), which recognizes and directs the nuclease to target DNA sequence, and a Cas9 nuclease for cleavage double strand DNA sequence^15–17^. The double strand breaks (DSBs) of target DNA are mainly repaired through the mutagenic non-homologous end joining (NHEJ), resulting in disruption and mutation of the targeted gene. Due to its high efficiency, ease, speed, and therefore relatively low expenditure, the CRISPR/Cas9 system has been successfully applied in a wide variety of organisms and shown great potential for rapidly generating somatic mouse models of certain human tumors^18–21^. Recently, Xue *et al*. Showed that hydrodynamic injection of the CRISPR/Cas9 system to target *p53* and *Pten* genes in adult mice could yield cancer-related phenotypes in liver after carbon tetrachloride treatment^22^. They showed that although hydrodynamic injection can deliver plasmid DNA to only about 20% of hepatocytes in mice, the CRISPR/Cas9–mediated genome editing is sufficient to induce multifocal tumors in adult mouse liver. More importantly, this method bypasses the need to engineer the mouse germline to create targeted mutant animals. Thus, the CRISPR/Cas9-mediated genome editing delivered by tail vein hydrodynamic injection provides an alternative approach to research the mechanism of tumorigenesis in liver in adult mice^22^.

In the present study, we investigated the liver tumorigenesis in HBV-transgenic mice by simultaneously introducing *p53* and *Pten* mutation through CRISPR/Cas9 system. We showed that CRISPR/Cas9 system delivered by hydrodynamic tail vein injection can be used to somatically induce *p53* and *Pten* mutation in the liver of HBV-transgenic mice, leading to the induction of HCC as early as 4 months’ post injection. Moreover, this study provided a fast and convenient method for generating mouse model of liver cancer with HBV infection characteristics.

## Results

### CRISPR/Cas9 system with dual sgRNA expressing cassette induced *p53* and *Pten* mutation *in vitro*

**Figure 1:**
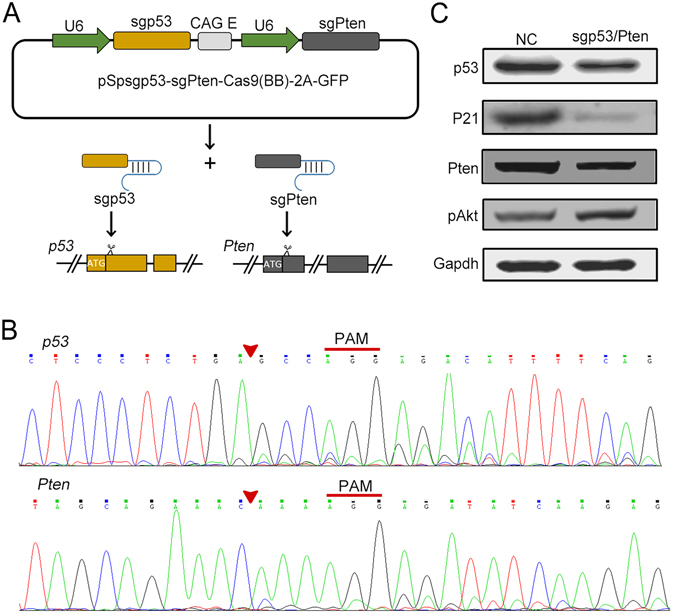
Dual sgRNA cassette induced loss-of-function alterations of *p53* and *Pten* genes in H2.35 cells. (**A**) Schematic for the dual sgRNA expressing cassette containing both *p53*-specific sgRNA and *Pten*-specific sgRNA. (**B**) Indel mutations of *p53* and *Pten* identified by sequencing in the sgp53/Pten dual cassette transfected cells. H2.35 cells were transiently transfected with dual sgRNA cassette plasmids. Forty-eight hours later, cellular genomic DNA in H2.35 cell pools were extracted and PCR sequencing assays were performed to analyze the indel mutations of *p53* and *Pten*. Red triangles indicate the predicted DNA cleavage sites. The proto-spacer adjacent motif (PAM) sequences were denoted by red lines. (**C**) Loss-of-function alterations of *p53* and *Pten* genes detected by Western blot assay. Gapdh was used as control.

To induce *p53* and *Pten* gene mutation simultaneously, we constructed a dual sgRNA cassette plasmid by inserting sgRNAs of *p53* and *Pten* into the pSpCas(BB)-2A-GFP (PX458) vector. The sgRNAs specific for *p53* or *Pten* were designed to target the first exon of mouse *p53* or *Pten* gene, respectively. As shown in Fig. 1A, this p53/Pten sgRNA (sgp53/Pten) dual cassette was expected to express Cas9 endonuclease and specific sgRNAs targeting *p53* and *Pten* genes. Each sgRNA was driven by an independent RNA Pol III U6 promoter and regulated by a cytomegalovirus early enhancer/chicken β actin/rabbit β globin (CAG) enhancer.

The CRISPR/Cas9-induced mutations usually occur around the cleavage sites three bases upstream of the proto-spacer adjacent motif (PAM) and repaired mainly by cells’ NHEJ, resulting in insertions or deletions (indels). To validate the activity of the sgp53/Pten dual cassette in editing the genomic *p53* and *Pten* loci, we transiently transfected the dual sgRNA cassette plasmids into H2.35 cell, an immortalized mouse liver cell line derived from BALB/c mouse. The cellular genomic DNA in H2.35 cell pools were extracted, amplified and sequenced for analyzing the indel mutations of *p53* and *Pten*. The results showed that the sgp53/Pten dual cassette induced mutations at the predicted cleavage sites in front of the PAM in both *p53* and *Pten* genes (Fig. 1B). Western blot assay further confirmed that the dual sgRNA cassette vectors could efficiently decrease the expression of *p53* and *Pten* protein (Fig. 1C). Coincident with the downregulation of p53, expression of p21 which was positively regulated by p53 decreased significantly in sgp53/Pten dual cassette transfected cells. Moreover, the expression of phospho-Akt (pAkt, S473), a biomarker of the PI3K pathway activity increased after the sgp53/Pten dual cassette administrated (Fig. 1C). Taken together, these results demonstrated that the sgp53/Pten dual cassette could successfully express *p53*-specific and *Pten*-specific sgRNA and lead to *p53* and *Pten* mutation simultaneously.

### Hydrodynamic injection of the sgp53/Pten dual cassette accelerated tumor formation

**Figure 2:**
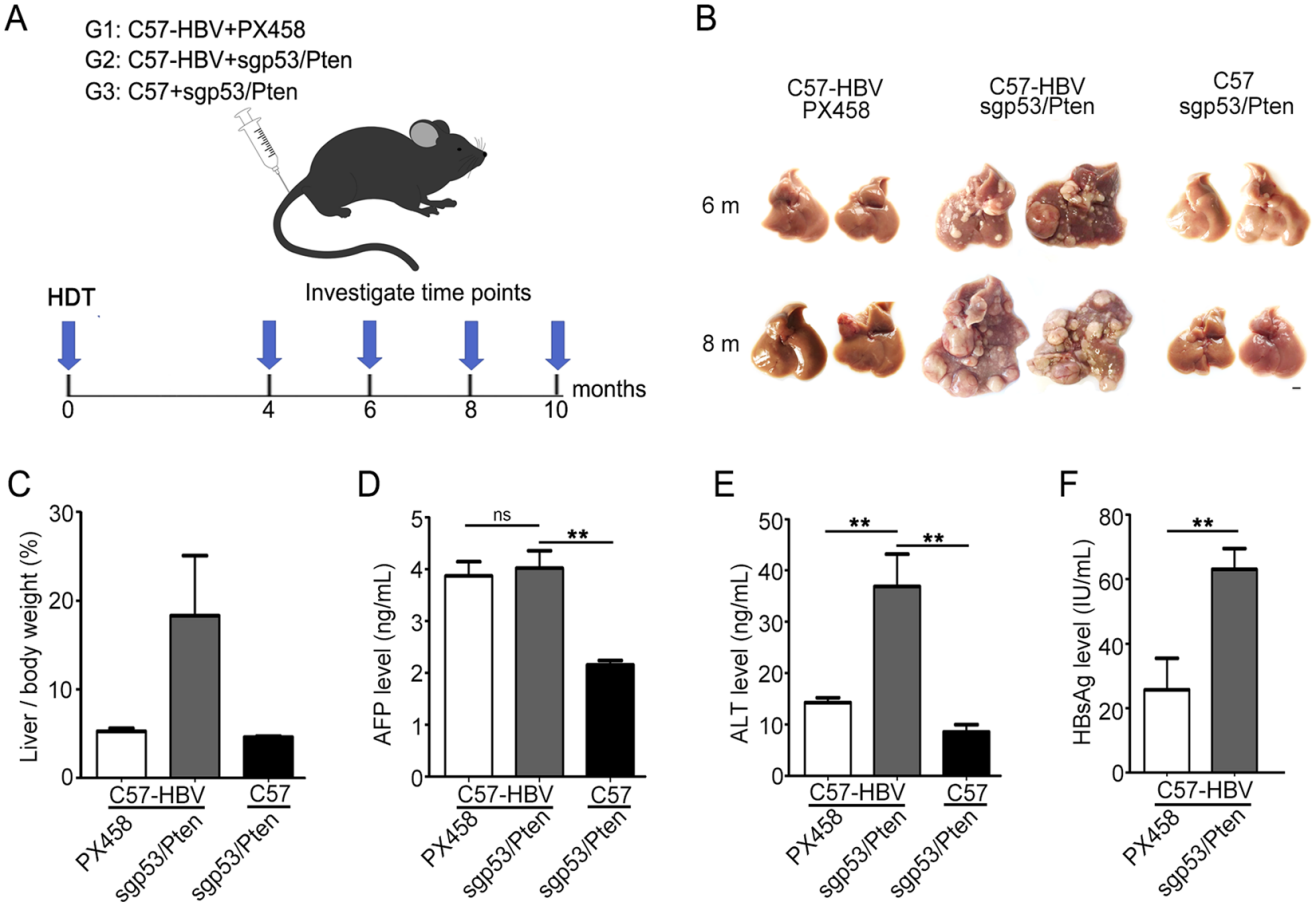
Hydrodynamic injection of dual sgRNA cassette accelerated tumor formation in HBV transgenic mice. (**A**) Cartoon showing hydrodynamic tail vein injection of dual sgRNA cassette to an adult mouse and the experimental design. HDT, Hydrodynamic tail vein injection. G1, group 1; G2, group 2; G3, group 3. (**B**) Liver tumors detected in the three groups of mice harvested 6 and 8 months’ post injection. Two representative photographs of mouse livers were showed in each group. At 6 months’ post injection, 3 mice in C57-HBV PX458 group, 3 mice in C57-HBV sgp53/Pten group, and 5 mice in C57 sgp53/Pten group were sacrificed. At 8 months’ post injection, the number of the sacrificed mice in the three groups were 3, 4 and 4 respectively. The scale bar is 5 mm. (**C**) Liver weight relative to the whole body weight of mice 8-month post injection. (**D** & **E**) Circulating AFP and ALT level in sera of the mice 8 months’ post injection with PX458 or dual sgRNA cassette. (**F**) HBsAg expression detection in sera of the C57-HBV mice 8-month post injection. Data represent means ± SEM (n ≥ 3). Statistical significance was determined with Student *t* two-tailed test. ** indicated P < 0.01.

To determine whether the *p53* and *Pten* mutation could induce liver tumorigenesis in transgenic mouse mimicking chronic HBV infection, we delivered the sgp53/Pten dual cassette plasmids to the liver of adult HBV transgenic C57 mice-Tg(HBV Alb-1)Bri44. This HBV transgenic mouse model had an inserted HBV DNA fragment containing the coding region for the HBV envelope polypeptides in its genome, and could express HBV large envelope polypeptide under the albumin promoter^23^. Viral protein expression in this model caused hepatocyte damage and liver necroinflammation at 4 months of age and induced macroscopic HCC nodules from 12–20 months^24^. The sgp53/Pten dual cassette plasmids were delivered to the livers of 5–7 week old C57-HBV mice by hydrodynamic tail vein injection. In parallel, a group of strain-, age-, and gender matched HBV transgenic mice injected with PX458 were used as control. Also, another group of wild-type C57BL/6 mice injected with sgp53/Pten dual cassette plasmids were used as control of non-HBV infection. (Fig. 2A).

To examine the tumorigenesis, C57-HBV mice injected with sgp53/Pten dual cassette mice were sacrificed at 4, 6, and 8 months’ post tail vein injection. Since previous studies have shown that liver-specific p53 knockout mice developed liver tumors only after 14 months^25^, the mice in the two control groups were sacrificed from 6 months’ post injection. Macroscopic tumors were observed as early as four months’ post injection in C57-HBV mice injected with sgp53/Pten dual cassette (Supplementary Figure 1A). The size of tumors significantly increased over time and tumor-burdened livers showed more and larger nodules at 6 and 8 months’ post injection (Fig. 2B). In contrast, none of the control animals developed any detectable nodules at 6 and 8 months’ post injection although one of the C57 mice treated with sgp53/Pten dual cassette plasmids developed a small liver tumor at 10 months (Supplementary Figure 1B and C). Quantification showed that the ratio of liver weight to body weight in HBV-C57 mice injected sgp53/Pten dual cassette plasmids was significantly higher than that in control mice at 8 months’ post injection (20% vs 5%) (Fig. 2C). This results demonstrated that somatic gene dysfunction using hydrodynamic tail vein injection to transiently express Cas9 and p53/Pten dual gRNAs is sufficient to accelerate tumor formation in HBV transgenic mice.

Serum AFP is the most widely used tumor marker in detecting patients with HCC. At the 8 months’ post injection, the serum AFP level in tumor burdened C57-HBV mice was significantly higher than that in C57 mice injected with sgp53/Pten dual cassette plasmids, but it made no difference to that in HBV-C57 mice injected with PX458 plasmids (Fig. 2D). We also detected serum ALT level, a hepatocyte damage marker in these three group animals and found that the ALT level was extremely higher in the serum of tumor harboring C57-HBV mice, as compared to that of the tumor-free C57 mice or C57-HBV mice (Fig. 2E). In parallel, the serum HBsAg level was also significantly elevated in the tumor harboring C57-HBV mice injected with sgp53/Pten dual cassette (Fig. 2F). Taken together, these results suggested that sgp53/Pten dual cassette treatment induced more serious liver damage in HBV transgenic mice.

### Pathology of liver tumors generated in HBV transgenic mice via sgp53/Pten dual cassette injection

**Figure 3:**
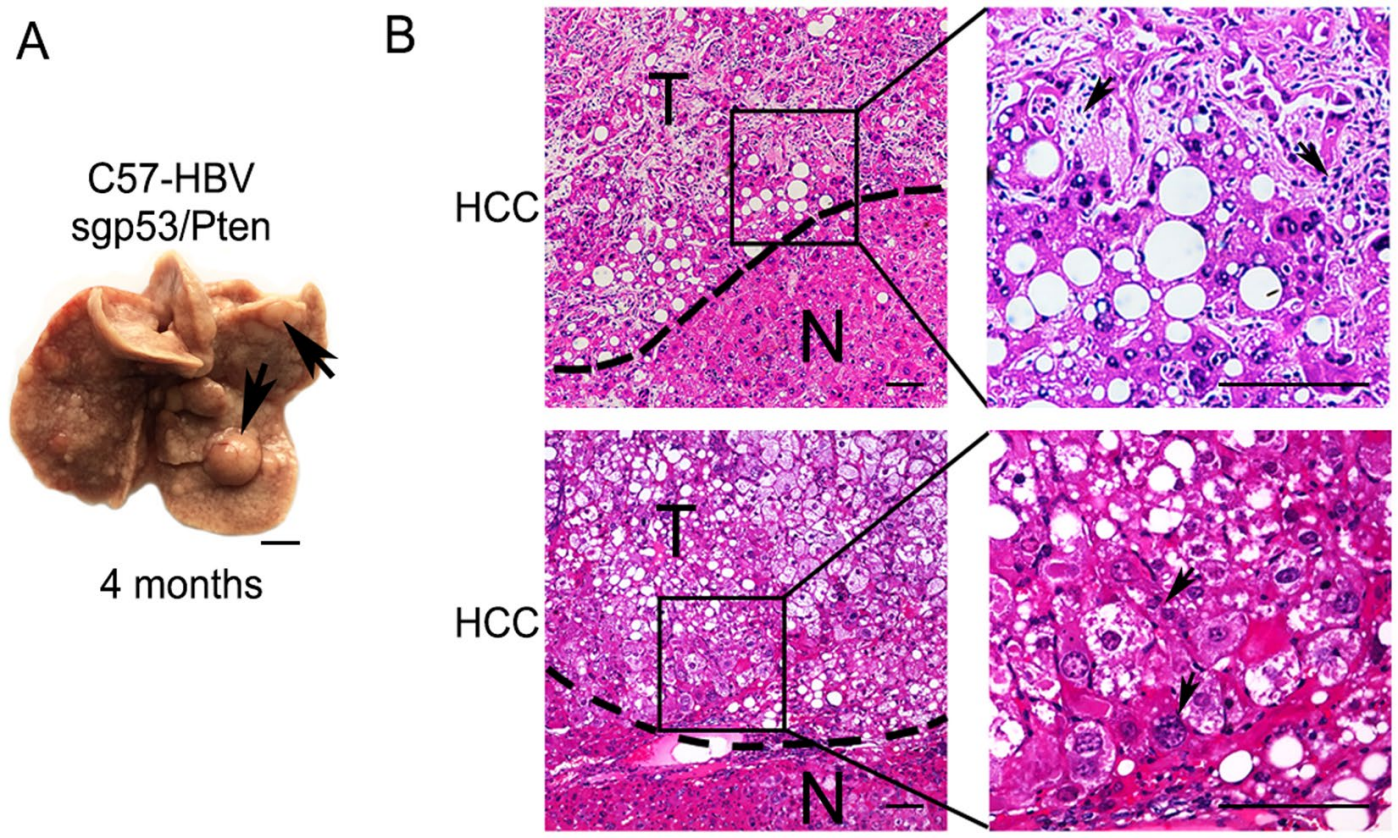
Liver tumors observed in C57-HBV mice with sgp53/Pten dual cassette. (**A**) Macroscopic view of representative liver tumors (arrows) observed in C57-HBV mice 4 months’ post injection. The scale bar is 5 mm. (**B**) H&E-stained section of the tumor (the small one in A) showing the HCC with lipid accumulation and a trabecular-like arrangement that disrupts normal liver architecture. H&E-stained section of the tumor (the big one in A) showing its cytological atypia such as huge nuclei and multinuclear hepatocytes and foamy, reticulated pattern in cytoplasm of HCC. T, tumor; N, non-tumor. The scale bar is 100 μm.

**Figure 4:**
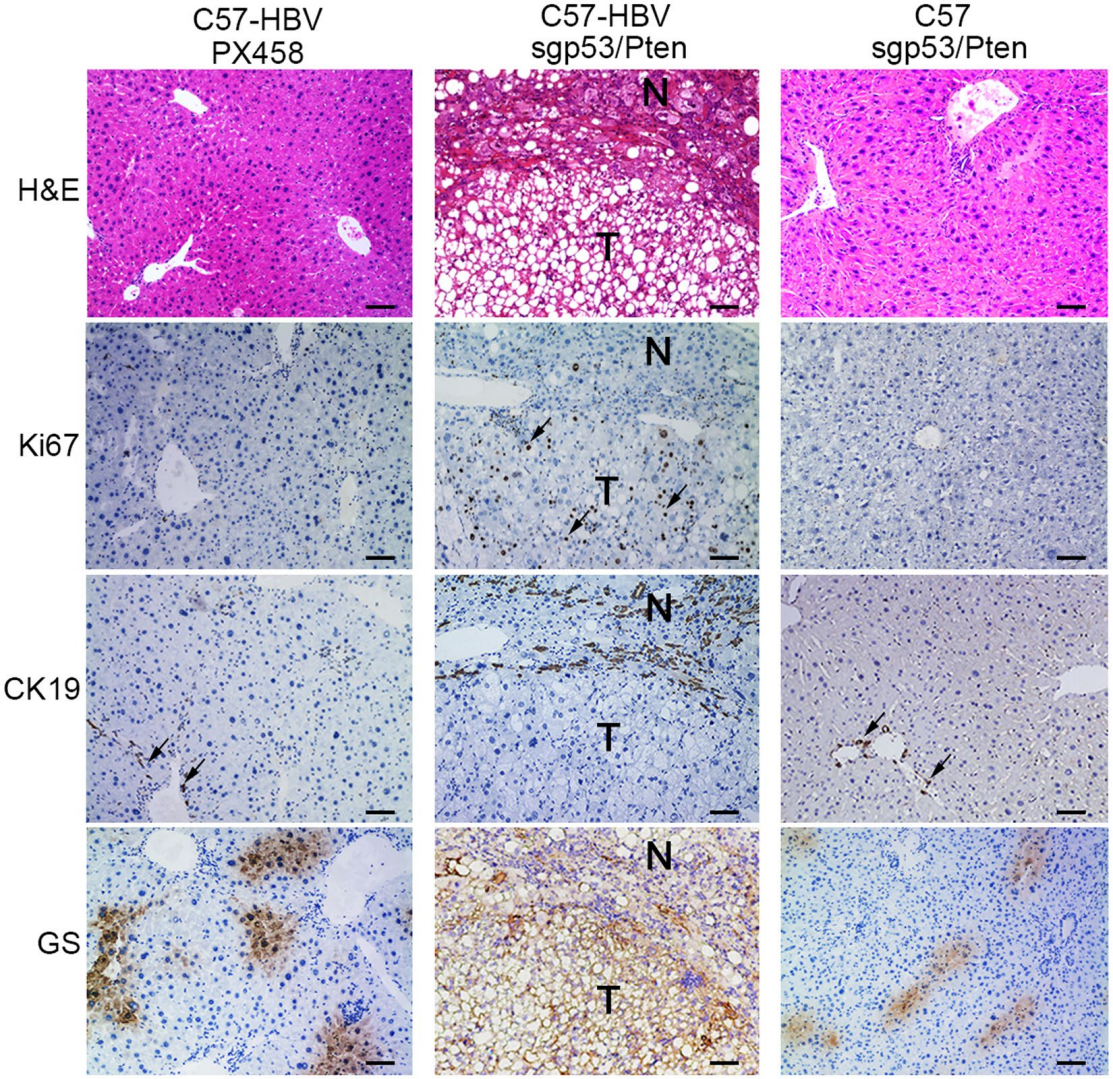
Histological and pathological determination. H&E staining, Ki67, CK19 and GS IHC staining of the liver sections in the three groups. For Ki67 and CK19 IHC staining the represent positive staining cells were showed by arrows. T, tumor; N, non-tumor. The scale bar is 200 μm.

To understand the pathology of tumors formed by injection of the sgp53/Pten dual cassette, we performed hematoxylin and eosin (H&E) staining and immunohistochemistry (IHC). The liver sections from the dual cassette-injected C57-HBV mice displayed nodules indicating HCC, which exhibiting obscured boundary from the surrounding non-tumor liver tissue (Fig. 3A and B). On the other hand, the nodules were composed of confluent areas of cytological atypia such as huge nuclei and multinuclear hepatocytes (arrows indicated) which in accordance with the character of primary carcinoma of the liver with significant lipid or glycogen accumulation in cytoplasm (Figs 3C and 4 first row). In contrast, none of the control mice showed any tumors detectable by histology.

To further confirm that the nodules were malignant proficiency, IHC of Ki67 was performed to indicate proliferating cells. The results showed that tumor cells were positive for Ki67, signifying a more rapid proliferation rate in these tumor cells as compared to that in adjacent non-tumor tissues (Fig. 4, second row). Next, Glutamine synthetase (GS),a marker of hepatocellular tumor, was used to identify the histological types of these liver tumors. The results showed that GS was diffusely positive throughout the nodules which support the characteristic of HCC (Fig. 4, last row). Furthermore, the staining of cytokeratin 19 (CK19), a marker of biliary lineage cells, showed no immunoreactivity to CK19 in the tumor tissues from the C57-HBV mice delivered with sgp53/Pten even though significant CK19 staining could be detected in the adjacent non-tumor tissues (Fig. 4, third row). The observation that the absence of biliary duct in these tumors excluded the origin of bilinear lineage cells and was consistent with the diagnosis of HCC. Taken together, these results demonstrated that delivery of sgp53/Pten dual cassette could induce HCC development in the livers of HBV transgenic mice as early as 4 months’ post tail vein injection.

### The sgp53/Pten dual cassette induced high frequency of *p53* and *Pten* mutation in the tumors of HBV transgenic mice

**Figure 5:**
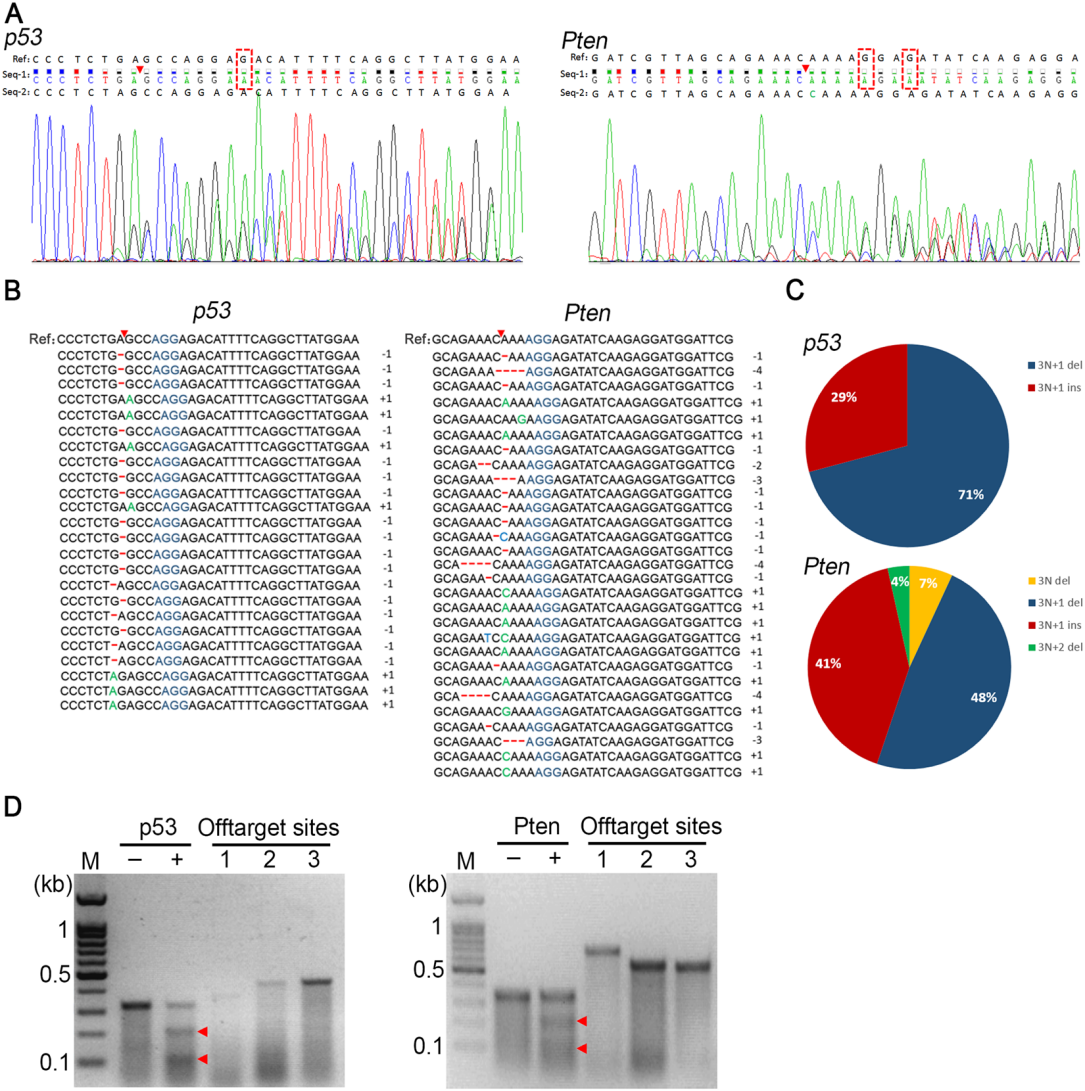
Mutational analysis of individual tumors of C57-HBV mice injected with dual sgRNA cassette. (**A**) Representative *p53* and *Pten* mutations found in the predicted cut sites in tumors. The reference sequences were mouse genomic sequences from the National Center for Biotechnology Information (NCBI; *p53*, genebank ID:372099099; *Pten*, genebank ID:372099091). Seq-1 and Seq-2 represent read sequences obtained from sequencing chromatograms. Different bases between Ref and Seq-1 were revealed with red dashed rectangles. Red triangles indicate the predicted cleavage sites. (**B**) Sequence analysis near the predicted cleavage sites of *p53* and *Pten* in the tumors. A total of forty tumor nodules were examined and only the mutant sequences of p53 or Pten were shown. Red triangles, predicted cleavage sites; Blue bases, the PAM motif; Red lines, deletion; Green bases, insertion; Bases in light blue, mutation. (**C**) Indel mutation profile of *p53 and Pten* in the tumors. Distribution of indel frame phase was calculated as the length of modulus 3. (**D**) Off-target assessment using the T7E1 nuclease. The *p53* and *Pten* top ranking off-target sites 1, 2, 3 were investigated. −, negative control; +, positive control. Red triangles indicate the bands cut by the T7E1 nuclease. M, marker.

We next sought to determine whether the sgp53/Pten dual cassette induced direct genome editing of *p53* and *Pten* genes in liver tumors. The tumor nodules were isolated from livers of C57-HBV mice which were collected at six and eight months after injection. The direct sequencing of the PCR products demonstrated the presence of indels in *p53* and *Pten* genes at the predicted cleavage sites in these tumor nudes (Fig. 5A). A large fraction of these indels potentially disrupted the endogenous gene function because they were mostly out of frame (3 *N* + 1 base pair insertion or deletion) (Fig. 5B and C). The frequency of *p53* and *Pten* dual mutation in tumor tissues was as high as 45% at 6 months(n = 20) and 75% at 8 months (n = 20) post injection, suggesting an increase of *p53* and *Pten* mutation frequency over time which implicated the selective growth advantage of mutant cells. In contrast, such indels of *p53* and *Pten* genes were not found in the control livers either from C57 mice injected with sgp53/Pten dual cassette or the C57-HBV mouse injected with PX458 plasmids (data not show).

To assess the potential off-target effects of sgp53/Pten dual cassette *in vivo*, we used T7E1 assay to identify the top ranking off-target gnomic sites for *p53* and *Pten*. We amplified top three potential off-target sites of *p53* or *Pten* loci from liver tumor with *p53* and *Pten* dual mutation. Also, *p53* and *Pten* loci were amplified from the liver treated with PX458 as the negative control. The T7E1 assay revealed a significant cleavage with exact cut products in the sgp53/Pten dual cassette treated liver tumors, while no T7E1 nuclease cutting was detected at the assayed off-target sites (Fig. 5D). These results suggested that the frequency of off-target editing was negligible, at least bellow the limit of detection in this assay.

### The sgp53/Pten dual cassette induced somatic dysfunction in the liver of HBV transgenic mice

**Figure 6:**
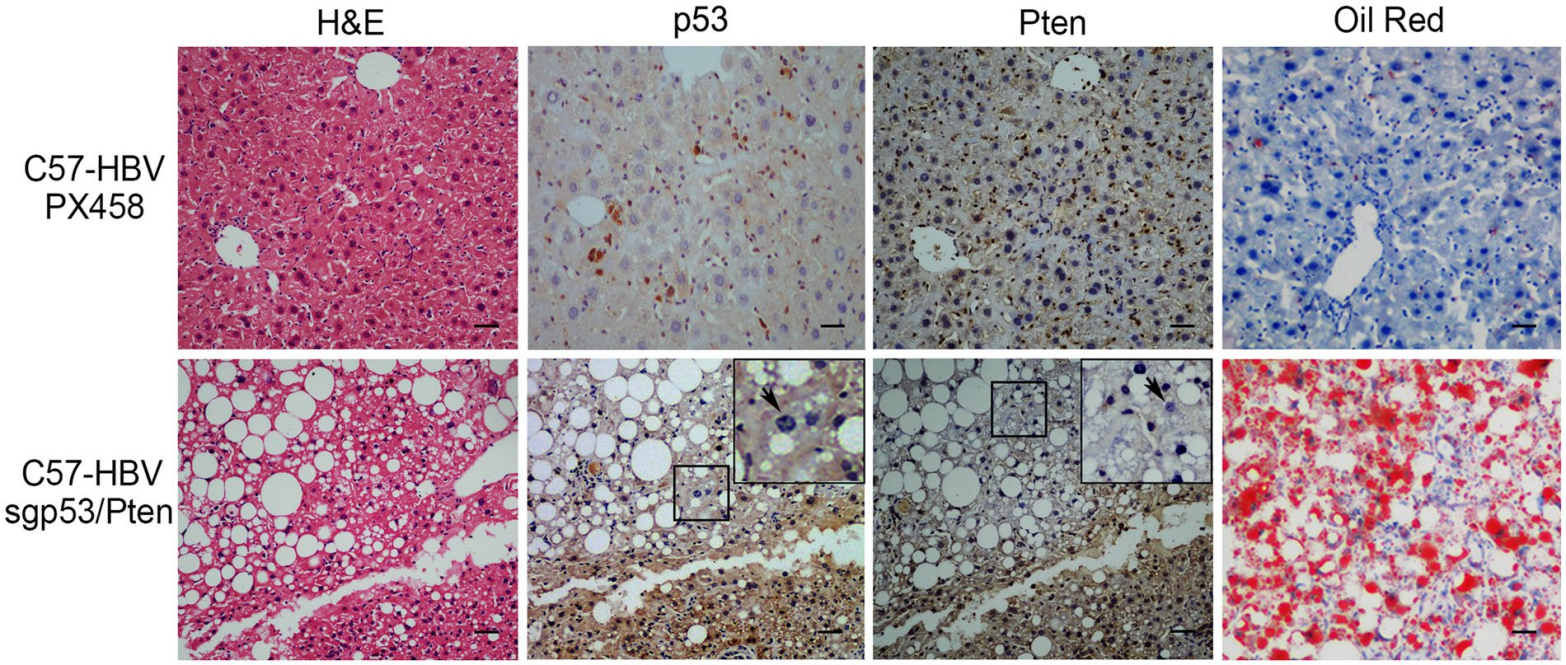
IHC assay of somatic dysfunction in liver tissues of HBV transgenic mice. Liver sections from C57-HBV mice injected with sgp53/Pten or PX458 (as a control) were strained by H&E, p53, Pten and Oil Red Staining The insets are high-magnification views (X400). The arrows in p53 and Pten IHC indicated the negative stained hepatocyte. The scale bar is 100 μm.

We next examined whether the indel mutations induced by CRISPR/Cas9 system could result in functional loss of p53 and Pten in liver tumors of HBV transgenic mice. The IHC assay revealed that in sgp53/Pten dual cassette injected C57-HBV mice, tumor tissues had a lower rate of p53 protein positively staining than that in the non-tumor region (Fig. 6). Similarly, the level of Pten protein in tumors was also significantly decreased in sgp53/Pten dual cassette injected C57-HBV mice, as compared to the surrounded non-tumor liver tissues and the normal liver tissues of PX458-C57-HBV mice (Fig. 6). It has been reported that deletion of Pten in the liver would result in increased fatty acid synthesis, accompanied by hepatomegaly and steatohepatitis^13, 14^. To assess if such phenotype was associated with Pten mutation, we stained liver sections of C57-HBV mice injected with PX458 and sgp53/Pten dual cassette with Oil Red. The livers of sgp53/Pten dual cassette-injected C57-HBV mice developed steatosis and had increased numbers of fat droplets in cytoplasm of hepatocytes (Fig. 6). These data indicated that *in vivo* CRISPR-mediated genome editing was able to induce loss-of-function mutations *of p53* and *Pten* and resulted in corresponding somatic dysfunction.

## Discussion

The manipulation of genes of interest has been vital for deciphering their roles within a cell or an entire organism. For the first time, we constructed a CRISPR/Cas9 system directly targeting the tumor suppressor genes *p53* and *Pten* in combination and delivered this system by hydrodynamic tail vein injection to the liver of HBV-transgenic mice. We demonstrated that the CRISPR/Cas9 mediated mutations of *p53* and *Pten* loci in adult mice were sufficient to accelerate HCC development in HBV-transgenic mice without treatment of any chemical carcinogen.

The role of HBV in tumor formation appears to be complex and may involve multiple pathways, including the accumulation of genetic damage due to immune-mediated hepatic inflammation, the induction of oxidative stress, the virus-specific mechanisms involving the HBV viral proteins X and S, and the integration of HBV DNA into the host genome^26^. Integration of HBV DNA into the host genome occurs at early steps of clonal tumor expansion, and induces both genomic instability and direct insertional mutagenesis of diverse cancer-related genes^27, 28^. Compared with tumors associated with other risk factors, HBV-related tumors have a higher rate of chromosomal alteration and gene mutation. These genomic alterations are suspected to play a major role in HCC carcinogenesis during HBV infection^5, 7, 29–32^. However, the functional importance and physiological impact of most tumor genetic alterations in HBV-related HCC remains poorly defined.

Previous studies have shown that this HBV transgenic mouse could generate macroscopic HCC from age 12–20 months, due to the coagulative necrosis and lobular macrophagic inflammation induced by accumulation of large HBV surface antigen (HBsAg) within the endoplasmic reticulum of the hepatocytes^23, 24^. In this study, we designed sgp53/Pten dual cassette system to target these two genes to induce indel mutations in the livers of adult HBV transgenic mice. The results showed that simultaneous mutation in *p53* and *Pten* loci induced HCC nodules in HBV transgenic mouse as early as 4 months, but failed to induce tumors in wild-type C57 mice even at 8 months’ post injection. Importantly, elevated serum HBsAg and ALT levels were seen in HBV transgenic mouse following sgp53/Pten dual cassette induced p53 and Pten deficiency in mouse livers. Since chronic micro-environmental inflammation is considered as a major causative factor for liver malignancy transformation in chronic HBV infected individuals, the loss-of-function mutations in *p53* and *Pten* might aggravate chronic liver inflammation and liver injury induce by HBsAg and thereby accelerating the tumor development of HCC. To the best of our knowledge, our study provided the first genetic evidence to support the postulation that mutation/deletion of p53 and Pten may act as cancer drivers in HBV-related HCC. In addition, we also noticed that the serum AFP level, a most commonly used tumor biomarker for HCC, was higher in C57-HBV mice no matter bearing tumor burden or not than that in C57 mice at 8 months’ post injection. In consistent with our observation, previous studies have reported that HBV could promote transcription of AFP by acting on the elements in the AFP gene regulatory region^33^. Clinical research also indicated that the positive rate of AFP and the median serum AFP level in HBV infection-related HCC patients were significantly higher than that in patients irrelevant to HBV infection^34^. Therefore, the current study provided a further experimental support for the involvement of HBV infection in elevating serum AFP.

Pten is a well-known negative regulator of the PI3K/AKT pathway and plays an import role in regulating cell survival, apoptosis and protein translation^35^. Pten loss results in constitutive activation of the PI3K/AKT pathway, leading to the transcription of genes involved in angiogenesis and survival^36, 37^. In a mouse model generated by the Cre-loxP system, the liver-specific knockout of Pten induced lipid accumulation, steatohepatitis, as well as adenomas or HCC at older age (74–78 weeks), suggesting that loss of Pten may participate in liver carcinogenesis^13, 14^. Studies of CRISPR/Cas9-mediated somatic genome editing of Pten in wild-type mice also demonstrated that Pten loss led to increased Akt phosphorylation and hepatic steatosis in hepatocytes at 4 months’ post treatment^22, 38^. Previous work has been confirmed that hepatic steatosis can progress to the more aggressive nonalcoholic steatohepatitis (NASH), which is distinguished by the presence of hepatocyte injury, infiltration of inflammatory cells, and may display collagen deposition^39–41^. The uncontrolled inflammation, cycles of necrosis and regeneration as displayed in NASH has been identified as one of the key events in enhancing hepatic carcinogenesis^42^. Consistent with literature data showing the critical roles of hepatic steatosis in tumorigenesis, we also observed that the tumor tissues exhibited a phenotype of significant lipid accumulation and obvious character of primary clear cell carcinoma of the liver (PCCCL) in HBV transgenic mice, which contained the CRISPR/Cas9-mediated Pten loss in livers. Since the expression of HBV viral protein such as HBsAg could also induce chronic immune-mediated liver cell injury, we speculated that the Pten loss-induced lipid accumulation in hepatocyte would aggravate the inflammation of the liver and thereby promote hepatocarcinogenesis during the chronic HBV infection.

p53 is a pivotal tumor suppressor which induces apoptosis, cell-cycle arrest and senescence in response to stress signals. A high frequency of p53 inactivation in HBV-HCC provides a potential pathogenic link between HBV infection and p53 inactivation, suggesting that HBV-related HCC underlying alternative mechanisms for tumorigenesis to some extent^5, 7, 8^. Recently, studies from Xue *et al*. had showed that in carbon tetrachloride-treated wild-type mice, the hydrodynamic injection of CRISPR/Cas9 system targeting *Pten* and *p53* induced liver tumors with bile duct differentiation features at 3 months’ post injection. However, when injected alone, neither sgPten nor sgp53 caused any detectable tumors at this time point^22^. This observation suggested that without the protective surveillance afforded by p53, Pten-deficient hepatocytes were more susceptible to the development of liver cancer. In addition, it has been reported that p53−/− tumors in mouse models exhibit a mixed differentiation with hepatocytic and cholangiocytic features and loss of p53 function was associated with stem cell-specific gene expression signatures in human HCC^25, 43^. Therefore, in the case of HBV infection, p53 loss may predispose to the initiation of hepatocyte tumors by inducing liver stem/progenitor cells to differentiate into hepatocytes. On the other hand, the chemical genotoxic drug may be more likely to make liver stem/progenitor cells differentiate to other subtype of liver cells and induce tumor with cholangiocytic features, as Xue *et al*. reported in their study^22^.

In summary, the current study provides the first experimental evidence that the simultaneous mutation of p53 and Pten dramatically accelerates the formation of HCC in HBV transgenic mice without any chemical carcinogen. Additionally, we present a new way for establishing mouse model of HCC with HBV infection characters rapidly, which allowed us to test the oncogenic properties of any single genes or genes in combination in HBV transgenic mice.

## Materials and Methods

### CRISPR vectors construction

The sgRNA/Cas9 dual expression vector pSpCas9(BB)-2A-GFP (PX458) was obtained from Addgene (Cambridge, MA). The sequences for sgRNAs targeting *p53* and *Pten* genes were shown in Supplementary Table 1 ^22^. The construction of sgp53/Pten was in three steps. First, *p53*-specific and *Pten*-specific sgRNA/Cas9 expression vectors were constructed by ligating the annealed oligo-nucleotides of *p53* and *Pten* into the PX458 plasmid digested with Bbs *I* enzyme, particularly. Then the *p53*-specific sgRNA/Cas9 expression vectors were linearized just after the U6 terminator site by PCR amplification using the primers of sgp53-v-F and sgp53-v-R. The products were digested with Dpn *I* followed by gel purification. Second, the *Pten*-specific single guide RNA cassette was PCR amplified with U6-Pten-F and U6-Pten-R using the *Pten*-specific sgRNA/Cas9 expression vector as the template. The CAG enhancer was amplified from the PX458 plasmid with P8-cag-e-F and P8-cag-e-R. Then the two segments produced from the above PCR were recombined together by PCR-driven overlap extension. The recombinant products contain CAG enhancer, U6 promoter and the *Pten*-specific single guide RNA cassette. Third, the purified recombinant products and linearized *p53*-specific gRNA/Cas9 dual expression vectors were ligated using the Lightening Cloning Kit according to manufacturer’s instructions (Boao, Beijing, China). The primers used for PCR amplification were shown in supplemental Table 2. All the constructed plasmids were confirmed by Sanger sequencing.

### Animal experiments

C57BL/6 and HBV transgenic mice in a C57BL/6 background were from Peking University Health Science Center and maintained under standard barrier conditions (pathogen-free conditions provided by plastic cages with sealed air filters) in the Department of Laboratory Animal Science of Peking University Health Science Center. All the animal experiments were approved by the animal ethics committee of Peking University Health Science Center and were performed in accordance with relevant guidelines and regulations. Vectors for hydrodynamic tail vein injection were prepared using the Endo-Free Maxi Kit (Qiagen, Hilden, Germany). For hydrodynamic injection, 60 μg plasmid DNA for each mouse was suspended in 2 ml sterile phosphate-buffered saline (PBS) was injected via the tail vein in 5~7 s into 5~7 weeks old C57BL/6 or C57-HBV mice as previous described^44^. At least 3 mice per group were administrated. The animals were monitored thereafter by body weight and sacrificed by cervical dislocation at the indicated time points for serum and liver isolation. Tumors tissues were frozen in liquid nitrogen for DNA extraction and fixed for immunohistochemistry assays.

### Cell culture and transfection

Mouse H2.35 cell line obtained from ATCC (ATCC® CRL-1995™) were maintained in Dulbecco’s Modified Eagle Medium (DMEM) supplemented with 5% fetal bovine serum (Gibco, Carlsbad, Calif) at 33 °C in a 10% CO_2_ atmosphere. For transfection assay, H2.35 cells were seeded in a 6-well plate at 3 × 10^5^ cells/well. Twenty-four hours later, cells were transfected with 3 μg sgp53/Pten dual cassette plasmid using lipofectamine 2000 (Life Technologies, Carlsbad, CA) according to the manufacturer’s instruction.

### Western blotting

Proteins were extracted from H2.35 cells using Radio Immunoprecipitation Assay(RIPA) protein lysis buffer. Protein samples were quantitated and 80 μg of each protein extract was loaded onto 12% sodium dodecyl sulfate-polyacrylamide gels, electrophoresed, and transferred onto nitrocellulose membranes (Amersham Biosciences, Uppsala, Sweden). The membranes were blocked with 5% dried milk in PBS buffer for 1 hours. After blocking, membranes were incubated with primary antibody (anti-p53, anti-Pten and anti-pAkt were from Cell Signaling, MA; anti-p21, Abcam, Cambridge, UK; anti-Gapdh, Medical & Biological Laboratories Co., Ltd., Nagoya, Japan) over night at 4 °C. After 3 times washing with PBS containing 0.1% Tween-20, the membranes were incubated with secondary antibodies conjugated to LI-COR IRDye for 1 hour at room temperature. Band signals on the membranes were detected using the Odyssey Imager (LI-COR Biosciences, Lincoln, Neb).

### Detection of serum marker

Serum Alanine aminotransferase (ALT) and alpha fetoprotein (AFP) levels were determined using Mouse ELISA Kits of ALT and AFP (DingSheng, Beijing, China). Serum HBV surface antigen (HBsAg) were detected by a time-resolved fluoroimmunoassay (TRFIA) according to manufacturer’s instructions (PerkinElmer, Waltham, MA) and the concentrations were calculated according to the standard curve.

### DNA extraction

Genomic DNA from H2.35 cells was extracted using QIAGEN DNA mini kit (QIAGEN, Hilden, Germany) in accordance with the manufacturer’s instruction. Genomic DNA from livers or tumor tissues was purified by QIAamp DNA FFPE Tissue Kit (QIAGEN, Hilden, Germany).

### CRISPR-induced mutation Assay

Genomic DNA from H2.35 cells or tumor tissues were amplified by PCR using unique primers designed to span the expected indel positions (Supplementary Table 3). The PCR products were gel purified and then sequenced by conventional Sanger sequencing. The samples with overlapped sequencing chromatograms just follow by the predicted cleavage sites at three bases upstream of the proto-spacer adjacent motif (PAM) were supposed with mutation. Off-target effects were predicted using http://crispr.mit.edu/ and were identified with T7E1 assay system as^45^. Primers for amplifying *p53* and *Pten* off-target sites were shown in Supplementary Table 3.

### Immunohistochemistry(IHC)

Liver tissues were fixed in 4% formalin overnight, embedded in paraffin, sectioned at 4 μm and stained with hematoxylin and eosin (H&E) for pathology. The Oil Red staining was performed as previous described to visualize lipids^46^. Liver sections were de-waxed, rehydrated and stained for standard IHC. The primary antibodies used for IHC include anti-Ki67 (abcam, 16667, 1:200), anti-GS (abcam,197024, 1:8000), anti-CK19 (abcam, 133496, 1:100), anti-p53 (Cell Signaling, 2545, 1:160), anti-Pten (Cell Signaling, 9559, 1:100). Three randomly chosen magnification fields were imaged from each mouse liver with at least 3 mice per group.

### Statistical analysis

Data are reported as mean ± SEM. Differences between means were obtained by Student *t* test using GraphPad Software (GraphPad, Inc., CA) and a *p*-value less than 0.05 was considered significant.

## Electronic supplementary material

Supplementary Tables and Figures
