# Supplementary material for: CRISPR/Cas9-mediated p53 and Pten dual mutation accelerates hepatocarcinogenesis in adult hepatitis B virus transgenic mice
Source: Sci Rep. 2017 Jun 5;7:2796. doi: 10.1038/s41598-017-03070-8 (PMC5459841; doi:10.1038/s41598-017-03070-8)
Supplement: Supplementary file 1 — Supplementary Tables and Figures [file 41598_2017_3070_MOESM1_ESM.pdf]

CRISPR/Cas9-mediated p53 and Pten dual mutation accelerates hepatocarcinogenesis in adult hepatitis B virus transgenic mice

Yongzhen Liu[1], Xuewei Qi[1], Zhenzhen Zeng[1], Lu Wang[1], Jie Wang[1], Ting Zhang[1], Qiang Xu[1], Congle Shen[1], Guangde Zhou[2], Shaomin Yang[3], Xiangmei Chen[1]*, Fengmin Lu[1]

[1]Department of Microbiology & Infectious Disease Center, School of Basic Medical Sciences, Peking University Health Science Center, Beijing 100191, P.R. China.

[2]Department of Pathology and Hepatology, Beijing 302 Hospital, Beijing 100039, P.R. China.

[3]Department of Pathology, Peking University Health Science Center, Beijing 100191, P.R. China.

Yongzhen Liu and Xuewei Qi contributed equally to the presented work.

***Corresponding Authors:** Xiangmei Chen, Department of Microbiology & Infectious Disease Center, School of Basic Medical Sciences, Peking University Health Science Center, 38 Xueyuan Road, Beijing 100191, P.R. China. Email: xm_chen6176@bjmu.edu.cn;

Supplementary Table 1. Oligo sequences for p53 and Pten sgRNAs.

Target gene	20nt sequence (5' to 3')	PAM	Strand
Pten	AGATCGTTAGCAGAAACAAA	AGG	+
p53	CCTCGAGCTCCCTCTGAGCC	AGG	+

Supplementary Table 2. Oligo sequences for the construction of p53/Pten sgRNA dual cassette(sgp53/Pten).

Primers	Sequence (5' to 3')
sgp53-v-F	gctagaaatagcaagttaaaataaggctagtccgtttttagcgcgtgcgccaattctgc
sgp53-v-R	aaaaaagcaccgactcggtgccactttttcaagttg
U6-Pten-F	caatgggagtttgttttggagggcctatttcccat
U6-Pten-R	gccttattttaacttgctatttctagctctaaaacaaaaaagcaccgactcgg
P8-cag-e-F	gtggcaccgagtcggtgctttttcgttacataacttacggt
P8-cag-e-R	atgggaaataggccctccaaaacaaactcccattg

Supplementary Table 3. Primer sequences.

Primers	Sequence (5' to 3')	Notes
Pten-F	GAGCCATTTCCATCCTGCAG	PCR for Pten target region
Pten-R	CTAGCCGAACACTCCCTAGG	
p53-F	TCTGTCCTCCATGTTCCTGG	PCR for p53 target region
p53-R	TTTCTCTCAGGCAAGGGGAG	
PtenOT1-F	TCAGACTTTCCGTGCAGTCT	Pten off-target site 1
PtenOT1-R	ACAAGCGGAAACATCCACAC	
PtenOT2-F	TGGACTCTGCTTTGAAGGCA	Pten off-target site 2
PtenOT2-R	ATAAGCCCTGGGTAAGAGCC	
PtenOT3-F	TCCCAGTTCTTCCCAACCTC	Pten off-target site 3
PtenOT3-R	ACACACGTTCAGTTTCTGCC	
p53OT1-F	ATTCAGGCCCTCATCCTCCT	p53 off-target site 1
p53OT1-R	GAAGCCATAGTTGCCCTGGT	
p53OT2-F	TGGGTGAGGGTTAGTCCCAT	p53 off-target site 2
p53OT2-R	GAAATCAAAGCCACGCAGCA	
p53OT3-F	GCTCCTTCAGTCTGGCTGTT	p53 off-target site 3
p53OT3-R	CCCCTCCCATCTCCACACTA	

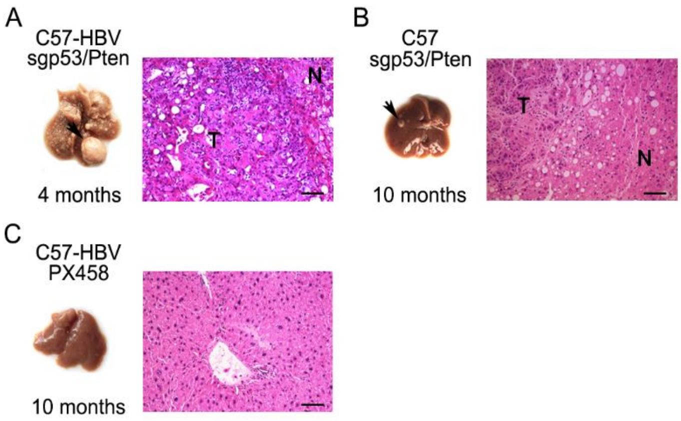

Supplementary Figure 1. Representative photographs of whole mouse livers and tumor sections detected by H&E staining of different groups in the indicated time points. (A) Macroscopic view of representative liver tumor (arrows) observed in C57-HBV mice 4 months' post sgp53/Pten injection. (B) Macroscopic view of representative liver tumor (arrows) observed in C57 mice 10 months' post sgp53/Pten injection. (C) The representative normal liver and section viewed in C57-HBV mice 10 months' post PX458 injection. T, tumor; N, non-tumor. The scale bar is 100 μm.
